# Working Conditions Supporting Person-Centered Care of Persons Living with Dementia in Long-Term Care Homes

**DOI:** 10.1093/geroni/igaa057.3516

**Published:** 2020-12-16

**Authors:** Marie Savundranayagam, Susan Docherty-Skippen, Shalane Basque

**Affiliations:** 1 Western University, London, Ontario, Canada; 2 Sam Katz Community Health and Aging Research Unit, London, Ontario, Canada

## Abstract

The COVID-19 pandemic has underscored the importance of person-centered dementia care and working conditions that support such care in long-term care (LTC) home settings. Personal support workers (PSWs), known also as certified nursing assistants, provide the most direct formal care for persons living with dementia. However, little is known about the working conditions that enable person-centered care. Accordingly, the purpose of this study was to examine the working conditions and the impact of those conditions on PSWs in LTC homes. PSWs (N=39) employed at one of five LTC homes in southwestern Ontario, Canada participated in a series of one-hour focus groups before, during, and after Be-EPIC, a person-centred communication training program for formal caregivers of persons living with dementia. Using an interpretive description investigative framework, textual data from focus group conversation transcripts were open-coded into categories. Overarching themes were interpreted inductively. Study credibility was enhanced through investigator triangulation. Three themes emerged related to working conditions of PSWs: dementia care is complex, lack of trained staff to provide person-centered dementia care, and residents’ families are not situated in the residents’ care circle. Four themes emerged related to the impact of current working conditions of PSWs: occupational burnout, poor resident care, frustrated and disengaged families, and PSWs leave their role. The findings offer opportunities for employers to ameliorate working conditions to support person-centered care. We conclude with specific workplace recommendations that respond to the complexity of dementia care and the associated occupational stresses PSWs experience in the current LTC environment.

